# Area-level poverty, race/ethnicity & dialysis star ratings

**DOI:** 10.1371/journal.pone.0186651

**Published:** 2017-10-17

**Authors:** Abhijit V. Kshirsagar, Raj N. Manickam, Yi Mu, Jennifer E. Flythe, Andrew I. Chin, Heejung Bang

**Affiliations:** 1 UNC Kidney Center, Division of Nephrology and Hypertension, University of North Carolina, Chapel Hill, North Carolina, United States of America; 2 Graduate Group in Epidemiology, University of California, Davis, Davis, California, United States of America; 3 Division of Nephrology, University of California, Davis School of Medicine, Sacramento, California, United States of America; 4 Division of Nephrology, Sacramento VA Medical Center, VA Northern California Health Care Systems, Mather Field, California, United States of America; 5 Division of Biostatistics, Department of Public Health Sciences, University of California, Davis, Davis, California, United States of America; 6 Center for Healthcare Policy and Research, School of Medicine, University of California, Sacramento, Sacramento, California, United States of America; Postgraduate Medical Institute, INDIA

## Abstract

The Centers for Medicare and Medicaid Services recently released a five star rating system as part of ‘Dialysis Facility Compare’ to help patients identify and choose high performing clinics in the US. Eight dialysis-related measures determine ratings. Little is known about the association between surrounding community sociodemographic characteristics and star ratings. Using data from the U.S. Census and over 6000 dialysis clinics across the country, we examined the association between dialysis clinic star ratings and characteristics of the local population: 1) proportion of population below the federal poverty level (FPL); 2) proportion of black individuals; and 3) proportion of Hispanic individuals, by correlation and regression analyses. Secondary analyses with Quality Incentive Program (QIP) scores and population characteristics were also performed. We observed a negligible correlation between star ratings and the proportion of local individuals below FPL; Spearman coefficient, R = -0.09 (p<0.0001), and a stronger correlation between star ratings and the proportion of black individuals; R = -0.21 (p<0.0001). Ordered logistic regression analyses yielded adjusted odds ratio of 0.91 (95% confidence interval [0.80–1.30], p = 0.12) and 0.55 ([0.48–0.63], p<0.0001) for high vs. low level of proportion below FPL and proportion of black individuals, respectively. In contrast, a near-zero correlation was observed between star ratings and the proportion of Hispanic individuals. Correlations varied substantially by country region, clinic profit status and clinic size. Analyses using clinic QIP scores provided similar results. Sociodemographic characteristics of the surrounding community, factors typically outside of providers’ direct control, have varying levels of association with clinic dialysis star ratings.

## Introduction

In 2014, the Centers for Medicare and Medicaid Services (CMS) introduced a Five-Star Quality Rating System as part of Dialysis Facility Compare (DFC) to help inform patients and caregivers in choosing a dialysis clinic in the United States (US). Released to the public in 2015 and updated in 2016, DFC uses a composite score generated from 8 individual clinical measures to quantify quality on a scale of 1 to 5 stars [1]: (1) standardized mortality ratio; (2) standardized transfusion ratio; (3) standardized hospitalization ratio; (4) adequate waste removal for hemodialysis; (5) adequate waste removal for peritoneal dialysis; (6) hypercalcemia; (7) percentage fistula; and (8) percentage catheters used >90 days. These measures are similar to those included in the more established End Stage Renal Disease (ESRD) Quality Incentive Program (QIP) [2].

While some of the measures are adjusted for case-mix, sociodemographic factors and other characteristics of the community surrounding the dialysis center are not considered. However, social determinants of health are known to contribute to disparities in outcomes in chronic kidney disease [3,4]. For example, it is plausible that inadequate community-level infrastructure and associated poor access to care may negatively influence quality measures. To date, there has been no assessment of the association of characteristics of the community surrounding dialysis clinics and dialysis clinic star rating scores.

In this paper, we examined the association of area-level poverty and other local population characteristics with dialysis clinic star ratings. Given the complexity of socioeconomic status and sociodemographic characteristics (e.g., race and ethnicity) in the US, a single benchmark may not be adequate [5]. Thus, we evaluated the relationship of the star ratings with the proportion of the population within the dialysis clinic census tract for each of the following: 1) below the federal poverty level (FPL); 2) black race; and 3) Hispanic ethnicity. Historically, black race and Hispanic ethnicity are two of the largest disadvantaged racial/ethnic groups in the US. Additionally, we examined the correlation of these 3 population characteristics with the dialysis star ratings by region of the country, dialysis clinic size and clinic profit status. In secondary analyses, we repeated the analyses with clinic QIP scores in place of star ratings. We hypothesized that area-level poverty and minority race/ethnicity, individually, inversely correlate with the DFC star ratings and QIP scores, and that differences in correlation strengths exist across U.S. regions, dialysis clinic sizes and profit status type.

## Materials and methods

### Study design and data sources

We used data from the Dartmouth Atlas that defines the dialysis facility area (year 2015) along with corresponding US Postal Zip codes [1]. Subsequently, geographical mapping was performed with Census Bureau Zip Code Tabulation Areas from the Uniform Data System Mapper, http://udsmapper.org/zcta-crosswalk.cfm. Data on poverty and race were retrieved from the American Community Survey by the Census Bureau, 5-year estimates in 2010–2014. The degrees of poverty and minority populations in a given census tract were determined by the percentage of the population below the FPL, percentage of black population, and percentage of Hispanic population.

Regions were categorized into Northeast, South, Midwest and West based on ESRD networks. Northeast included Networks 1,2,3, and 4; South included Networks 5,6,7,8,13, and 14; Midwest included Networks 9,10,11, and 12; and West included Networks 15,16,17, and 18. Size of each facility, data available on the DFC website, was categorized into Small, Medium and Large based on the number of total stations (using tertiles; cut-off points of 12 and 20). For-profit or non-profit designation was also retrieved from the DFC website, https://data.medicare.gov/data/dialysis-facility-compare.

Three of the seven individual metrics used to calculate the star ratings were adjusted for case-mix. Standardized mortality ratio, standardized hospitalization ratio, and standardized transfusion ratio were adjusted for the following patient characteristics at the level of the dialysis clinic: patient age, race, ethnicity, sex, diabetes, duration of ESRD, nursing home status, patient comorbidities at incidence (atherosclerotic heart disease, other cardiac disease, diabetes, congestive heart failure, inability to ambulate, chronic obstructive pulmonary disease, inability to transfer, malignant neoplasm, cancer, peripheral vascular disease, cerebrovascular disease, current smoker, alcohol dependence, and drug dependence), calendar year, body mass index at incidence [6–8]. The institutional review board at the University of California, Davis, determined the study to be exempt from full IRB review. We included raw data; see S1 File.

### Statistical analyses

Descriptive statistics (including mean, standard deviation, median, inter-quartile range (IQR)) were used to summarize the variables. Spearman correlation coefficients were computed between the DFC star rating and each of the sociodemographic measures (as covariates). All analyses were based on non-missing data; i.e., no imputation was employed. We used simple (bivariate) correlation, unadjusted for other covariates as we were interested in the correlations with ‘final, publicly available’ outcomes that already adjusted for case-mix and other factors. We computed correlation in the entire sample and within different strata/subgroups, pre-defined based on region, profit status, and size.

We used star rating as primary outcome and QIP’s total performance score as secondary outcome [2]. Both are integer-based scores with star ratings ranging 1–5 and QIP scores ranging 0–100. For both, a higher value is better. See the DFC website, references [2] and Table 1 for more information.

**Table 1:** Comparison of star rating and Quality Incentive Program Measures for payment year 2016.

Star Rating Measures	Quality Incentive Program Measures
Adequate waste removal (adult hemodialysis, pediatric, peritoneal dialysis)	Adequate waste removal (adult hemodialysis, pediatric, peritoneal dialysis)
Percentage fistula	Percentage fistula
Percentage catheters used >90 days	Percentage catheters used >90 days
Hypercalcemia	Hypercalcemia
Standardized Mortality Ratio	Blood stream infections
Standardized Hospitalization Ratio	Hemoglobin >12 g/dL
Standardized Transfusion Ratio	Reporting measures of anemia
	Reporting measures of mineral bone metabolism
	ICH-CAHPS patient satisfaction survey

ICH-CAHPS: In-Center Hemodialysis, Consumer Assessment of Healthcare Providers and Systems

Line graphs were drawn to illustrate the overall relationships between the outcome and key covariates. Simple and multiple linear regressions were fitted to associate the covariates (i.e., percentage of population below the FPL, percentage of black individuals, percentage of Hispanic individuals, profit status, region and size) with the number of stars in order to assess relative importance, impact of collinearity and robustness of associations (e.g., direction, magnitude, and statistical significance).

Ordered logistic regression was performed to assess the association of star ratings and sociodemographic characteristics to account for the ordinal nature of the star ratings. We used SAS 9.4 (SAS Institute, Cary, NC, USA) for data set-up and analyses.

## Results

Data for 6,032 dialysis clinics were available for analysis. Summary statistics of clinic and community characteristics are presented in Table 2. The mean star rating was 3.34 with a median of 3 (IQR of 3–4) and the median QIP was 60 (IQR 62–76). The Spearman’s correlation coefficient between star rating and QIP score was 0.60. The median proportion of individuals below the FPL was 16.6%, and the median household income was $46,950. The percentage of black and Hispanic individuals had median values of 7.9 and 7.4%, respectively.

**Table 2:** Dialysis facility rating and surrounding area-level characteristics.

Variable	N	Mean (SD)	Median (IQR)
**Facility Star Rating (primary outcome)**	6032	3.34 (1.1)	3 (3–4)
**QIP Score (secondary outcome)**	5998	68.7 (11.3)	69 (62–76)
**Percentage Below Federal Poverty Level**	6628	18.25 (10.2)	16.6 (10.5–23.8)
**Household Income (US $)**	6627	51,599 (20,360)	46,950 (37,788–61,346)
**Percentage Black Population**	6634	17.7 (22.1)	7.9 (2.4–24.6)
**Percentage Hispanic Population**	6634	16.6 (21.3)	7.4 (3.1–20.8)

SD: standard deviation; IQR: interquartile range; QIP: Quality Incentive Program. QIP file available in the Dialysis Facility Compare website includes 6245 records/provider IDs but 5998 have non-missing QIP scores.

Overall, there was a negligible inverse correlation between the dialysis star rating and percentage of the population below the FPL, correlation coefficient, R = -0.09 (p<0.0001); see Table 3. The correlation of star rating and the percentage of black population was R = -0.21 (p<0.0001). The correlation between star rating and percentage of Hispanic individuals was weaker; R = 0.04. The average percentage of population below the FPL in 1 to 5 stars was 19.7, 20.4, 18.4, 17.5 and 17.0%, respectively. The average percentage of black individuals was 22.3, 22.8, 19.2, 15.8 and 11.1%, respectively; see S1 Fig.

**Table 3:** Correlation of dialysis facility rating with measures of surrounding community.

	Percentage Below Federal Poverty Level	Percentage Black Population	Percentage Hispanic Population
**Dialysis Star Rating, Overall**	-0.09	-0.21	0.04 (0.001)
**Regions**			
**• Midwest (N = 1490)**	-0.11	-0.28	0.06 (0.02)
**• Northeast (N = 885)**	-0.18	-0.15	-0.04 (0.27)
**• South (N = 2563)**	-0.04 (0.04)	-0.08	0.006 (0.77)
**• West (N = 1072)**	0.02 (0.5)	-0.23	-0.12 (0.0001)
**Profit Status**			
**• For Profit (N = 5264)**	-0.09	-0.19	0.04 (0.002)
**• Not For Profit (N = 746)**	-0.08	-0.33	0.04 (0.24)
**Size**			
**• Small (N = 1502**^a^**)**	0.009 (0.72)	-0.26	-0.003 (0.91)
**• Medium (N = 2497)**	-0.08	-0.17	0.07 (0.004)
**• Large (N = 2011)**	-0.13	-0.17	0.09

If P≥0.0001, actual p-value is indicated in the parenthesis; If P<0.0001, p-value is omitted. P-values are unadjusted. With adjustment for multiple comparisons, a conservative threshold can be used for significance, p = 0.0017 (e.g., 0.05/30). P-value should be interpreted with caution when sample sizes are different.

^a^ 429 had missing/unreported data in star ratings.

The correlation between clinic star rating and area-level poverty and race composition differed according to other characteristics (Table 3). The correlation strength varied by region of the country. In the Northeast, R = -0.18 (p<0.0001) for the star rating and poverty, while in the West, a near null value was observed, R = 0.02 (p = 0.5). Among small clinics, correlation with poverty was R≈0.0 (p = 0.72), while R = -0.13 (p<0.0001) among large clinics. With regard to star rating and black population, the South showed the lowest correlation and the Midwest/West showed the highest correlation in the absolute scale (R = -0.08 vs. -0.28/-0.23, respectively). Reported profit status of the dialysis clinic also affected the correlation strength between start rating and race: R = -0.33 among non-profit clinics and -0.19 among for-profit clinics. The correlations between star ratings and percentage of black population were generally greater than correlations between star ratings and percentage of population below FPL as well as between start ratings and percentage of Hispanic individuals (p<0.0001 overall and within all subgroups).

Table 4 summarizes the unadjusted (Simple) and adjusted (Multiple) linear regression models with the star rating as continuous outcome and the five covariates. Collinearity appeared to be modest or negligible in that direction of the association was unchanged except for one coefficient near 0, the null value. The percentage of black population yielded a stronger, monotonic association with the outcome, compared to poverty (p<0.0001 for both of middle vs. low and high vs. low percentage, with difference of ≥0.30 point in average scores of star ratings). Poverty and size of clinic exhibited some nonlinearity, with smaller differences in scores (0.01–0.21 in absolute scale), which are also demonstrated in S1 Fig. Non-profit clinics had slightly higher scores on average (difference of 0.09 with p = 0.03) but when other factors were adjusted in the model, the difference was smaller and no longer statistically significant (0.03 with p = 0.44). Regions of Midwest, Northeast and South showed comparable ratings, all significantly lower (approximately -0.30, p<0.0001) than the West as referent. Also, large clinic size was associated with a lower rating, while middle vs. small size comparison showed statistically non-significant differences (p>0.25).

**Table 4:** Simple and multiple regression with outcome of the number of stars (N = 6032).

**a. Linear regression**		
**Factor**	Mean difference [95% CI] (p-value)	Adjusted Mean difference [95% CI] (p-value)
**% of poor population**		
**Mid vs. Low**	0.01 [-0.06, 0.07] (0.80)	0.06 [-0.01, 0.13] (0.07)
**High vs. Low**	-0.19 [-0.25, -0.12]	-0.05 [-0.13, 0.02] (0.13)
**% Black population**		
**Mid vs. Low**	-0.36 [-0.43, -0.29]	-0.30 [-0.37, -0.23]
**High vs. Low**	-0.48 [-0.54, -0.42]	-0.34 [-0.42, -0.26]
**Non-profit vs. profit**	0.09 [0.01, 0.17] (0.03)	0.03 [-0.05, 0.11] (0.44)
**Region: MW vs. W**	-0.33 [-0.41, -0.25]	-0.31 [-0.39, -0.23]
**NE vs. W**	-0.37 [-0.46, -0.28]	-0.30 [-0.39, -0.20]
**S vs. W**	-0.46 [-0.53, -0.38]	-0.29 [-0.38, -0.21]
**Number of stations**		
**Mid vs. Small**	-0.04 [-0.11, 0.03] (0.26)	0.03 [-0.04, 0.10] (0.44)
**Large vs. Small**	-0.21 [-0.28, -0.14]	-0.14 [-0.21, -0.06] (0.0003)
**b. Ordered logistic regression**		
**Factor**	Odds ratio [95% CI] (p-value)	Adjusted Odds ratio [95% CI] (p-value)
**% of poor population**		
**Mid vs. Low**	1.03 [0.92, 1.15] (0.66)	1.12 [1.00, 1.26] (0.05)
**High vs. Low**	0.72 [0.64. 0.80]	0.91 [0.80. 1.03] (0.12)
**% Black population**		
**Mid vs. Low**	0.53 [0.47, 0.59]	0.59 [0.53, 0.67]
**High vs. Low**	0.43 [0.38, 0.48]	0.55 [0.48, 0.63]
**Non-profit vs. profit**	1.18 [1.03, 1.35] (0.02)	1.07 [0.93, 1.23] (0.35)
**Region: MW vs. W**	0.55 [0.48, 0.64]	0.57 [0.49, 0.66]
**NE vs. W**	0.53 [0.45, 0.62]	0.60 [0.51, 0.70]
**S vs. W**	0.45 [0.39, 0.51]	0.59 [0.51, 0.68]
**Number of stations^+^**		
**Mid vs. Small**	0.87 [0.77, 0.97] (0.01)	0.97 [0.86, 1.09] (0.61)
**Large vs. Small**	0.65 [0.58, 0.74]	0.73 [0.65, 0.83]

If P≥0.0001, actual p-value is indicated in the parenthesis; If P<0.0001, p-value is omitted. High vs. Mid vs. Low were categorized using tertiles. CI: confidence interval.

When we repeated the analyses with the QIP score (Table 1) in place of star rating, we reached qualitatively similar results. For example, the correlations with percentages of the population below the FPL, black individuals and Hispanic individuals were -0.09 (p<0.0001) and -0.21(p<0.0001) and 0.02 (p = 0.19), respectively. In addition, when we fitted the ordered logistic models for star ratings, differences in effect sizes were more pronounced across clinic characteristics. For high and middle level of proportion black, the odds of higher rating versus lower rating were ~0.57 (p<0.0001) times lower than for low level of proportion black, given the other variables were held constant. Similar decreases in odds were observed for each of the three regions vs. West (odds ratio (OR) = 0.57–0.60, p<0.0001). In comparison, the proportion below FPL yielded reduced and inconsistent results (e.g., OR = 0.91 and 1.12, p≥0.05).

## Discussion

To our knowledge, no previous study has examined the association between the surrounding community characteristics and the dialysis star ratings and QIP scores. Programs like the DFC star rating system are part of The National Quality Strategy (NQS) to encourage effective public reporting for improving health care [9]. Patients, the target audience for the NQS, are both direct consumers of healthcare and drivers for improvement through choice. The impact of the surrounding community, via adjustment or stratification, is not typically factored in these programs over the concern that it may allow clinics providing inferior care to remain under-achieving. Rather, the goal of these programs is to raise all clinics to a high level of care.

Despite recent findings that the proportion of ESRD patients living in communities with poverty is high, our data suggest a weak correlation between dialysis star ratings and income-based poverty measures [10]. From a clinical and policy perspective, these findings suggest that there is perhaps a negligible impact of the surrounding community on clinic star ratings. The majority of dialysis patients have Medicare as their primary insurance since the passage of public law 92–603 in 1972 [11]. Medicare has provided near universal access to dialysis services for citizens of the US; as such, the ESRD program has been a force to reduce variations in access to care, ostensibly by combining insurance with delivery of comprehensive, clinically appropriate care [12,13].

On the other hand, these data demonstrate complexity with regard to the influence of the surrounding community on clinic quality scores. When race composition of surrounding communities is examined, there is stronger, albeit modest, inverse association with the star ratings with black race. For example, we found a surrounding community population of black race of 22% in clinics with the lowest star rating versus 11% in the highest star rating. The direction and absolute value of correlations remain consistent across most regions, clinic sizes, and profit status types suggesting that even with closer to universal access to health care via Medicare, differences by race might still exist. In contrast, we observed negligible associations of poverty status and Hispanic ethnicity with star ratings. Such findings are similar to those previously demonstrated for other quality metrics [14–16].

Also, previous studies have demonstrated differences by race in Medicare beneficiaries; one study showed higher than expected mortality in dialysis facilities that served large numbers of black patients [17], while another study in a different Medicare population demonstrated that black beneficiaries had increased mortality and lower use of services [18]. In other single payer systems with universal coverage like the Veterans Affairs system, similar black-white disparities exist, though these differences may be decreasing [19,20]. Surprisingly, the strongest correlation coefficient (in absolute value) appeared in areas that traditionally have a low proportion of blacks—the Midwest and West, while the correlation was weaker in the South (>0.2 vs. <0.1 in absolute value). We speculate a degree of neighborhood segregation by race might play a role in our findings; however, further delineation of this issue was beyond study scope [21].

Over the last decade, participants in the ESRD program have been at the forefront of the transition from traditional fee-for-service to value-based care. As such, dialysis providers and networks have faced an environment of pay-for-performance, including expanded capitated reimbursement (the ESRD Prospective Payment System) and a QIP with quality measures updated every few years [2, 22,23]. While the goal of the DFC star rating system is to promote informed patient choice, it may affect payments in two ways. First and most directly, clinics may lose patients in a market where other choices exist, especially in high population metropolitan areas. However, the extent of loss may be tempered by recent data showing consolidation of dialysis clinics and perhaps less choice [24]. Second, the star rating metrics have considerable overlap with those of the QIP. Dialysis clinics receive penalties of up to 2% of payment for services for failing to meet national or within-facility improvement targets in the QIP. Vascular access, anemia, bone and mineral metabolism, and dialysis adequacy represent measures in both DFC and QIP. Of these 4 areas, anemia management may be controlled by providers and dialysis facility decisions regarding erythropoiesis stimulating agents and iron administration. The other three–vascular access, bone and mineral metabolism, and dialysis adequacy–may be more susceptible to the impact of community infrastructure such as limited food choices and lack of specialty surgical care [25–29]. Furthermore, the type of vascular access leads to down-stream effects that also determine QIP metrics, including adequacy, catheter rates, hospitalizations and mortality [30,31].

The limitations of this study should be noted. First, our study was cross-sectional so we cannot easily distinguish effects from exposures, confounders and mediators. Second, our analyses were minimally adjusted as we did not have access to patient-level data. Yet, patients may use essentially the same set of limited variables to understand the quality of dialysis clinics at the DFC websites, similar to other rankings or rating statistics for consumers. CMS also uses these variables to assess payments. Reassuringly, our findings were robust across outcomes (star ratings and QIP scores) and different statistical methods. We hope this study may spur additional investigation with granular community data to provide better understanding of the impact of social determinants on DFC and other quality measures. Third, our study addresses numerical correlations and associations, not causation. However, it is widely perceived that sociodemographic and economic status is an important social determinant of health outcomes [32]. Fourth, we merged and analyzed publically available data, so we could not investigate the reasons for missing data and impact of possible selection bias. However, the missing data of star ratings and QIP scores were higher in non-profit and small facilities and lowest in the West region based on descriptive statistics; See S1 Table for more information. Finally, we used a single measure of FPL for all states–for a family of 4 in 2016, $24,300 per year. While this measure is the most widely used and updated for inflation using the Consumer Price Index, the FPL has different implications for different regions of the country. In areas with very high cost of living, this would pose a greater challenge than less costly regions, and may underestimate the impact of poverty on star ratings. Along the same line, zip codes are not perfect proxies for the sociodemographic characteristics of neighborhoods; homogeneity within a zip code is not necessarily the case.

## Conclusion

In summary, the DFC star rating is a new program meant to facilitate patient choice and reduce variation in the quality of care at dialysis clinics. This study suggests some characteristics of the community surrounding the dialysis clinic might influence the ratings, such as proportion of individuals of black race and geographic region in the country. We also identified factors that seem to have minimal influence on the rating (e.g., poverty, Hispanic, profit status and size of clinic). The practical implications of the observed effect sizes (the degree of correlation, odds ratio, difference in star rating) are unclear given some weak associations; thus more discussions are warranted. Additional analyses using more granular data are needed to more fully understand the influence of local area characteristics on star ratings and other clinical quality measures in dialysis. As healthcare in the US continues to move towards value-based purchasing and the incorporation of the patient voice, it is vital to understand the execution of programs like DFC in real-world settings.

## Supporting information

S1 Fig Star ratings in different subgroups. a) Percents of poor and black populations per star ratingb) Star rating per percent poor (below Federal Poverty Level) or black population;c) Star rating per dialysis clinic profit status (P = for profit; NP = not for profit) and size; andd) Star rating per region.
(DOCX)

S1 Table Characteristics in groups with missing vs. non-missing star rating. a) by profit status;b) by size;c) by region; andd) for continuous variables (poverty-level, % of black, % of Hispanic, median income).
(DOCX)

S1 File Raw dataset. (SAS7BDAT)
