# Supplementary material for: Area-level poverty, race/ethnicity & dialysis star ratings
Source: PLoS One. 2017 Oct 17;12(10):e0186651. doi: 10.1371/journal.pone.0186651 (PMC5645143; doi:10.1371/journal.pone.0186651)
Supplement: S1 Table — a) by profit status;b) by size;c) by region; andd) for continuous variables (poverty-level, % of black, % of Hispanic, median income). (DOCX) [file pone.0186651.s002.docx]

| \| \| \|  \| \| --- \| \| \| --- \| --- \| \| \| \| **The FREQ Procedure** \| \| --- \| \| \| --- \| --- \| \| \| \| **Table of Miss_star by OWNTYPE** \| \| \| \| \| \| --- \| --- \| --- \| --- \| --- \| \|  \| \| **OWNTYPE** \| \| **Total** \| \| **Non-Profit** \| **Profit** \| \| **Miss_star** \|  \| 757 \| 5275 \| 6032 \| \| **0** \| **Frequency** \| \| **Percent** \| 11.38 \| 79.29 \| 90.67 \| \| **Row Pct** \| 12.55 \| 87.45 \|  \| \| **Col Pct** \| 85.73 \| 91.42 \|  \| \| **1** \| **Frequency** \| 126 \| 495 \| 621 \| \| **Percent** \| 1.89 \| 7.44 \| 9.33 \| \| **Row Pct** \| 20.29 \| 79.71 \|  \| \| **Col Pct** \| 14.27 \| 8.58 \|  \| \|  \|  \| 883 \| 5770 \| 6653 \| \| **Total** \| **Frequency** \| \| **Percent** \| 13.27 \| 86.73 \| 100.00 \| \| \| --- \| --- \| --- \| --- \| --- \| --- \| --- \| --- \| --- \| --- \| --- \| --- \| --- \| --- \| --- \| --- \| --- \| --- \| --- \| --- \| --- \| --- \| --- \| --- \| --- \| --- \| --- \| --- \| --- \| --- \| --- \| --- \| --- \| --- \| --- \| --- \| --- \| --- \| --- \| --- \| --- \| --- \| --- \| --- \| --- \| --- \| --- \| --- \| --- \| --- \| --- \| --- \| --- \| --- \| --- \| --- \| --- \| --- \| --- \| --- \| \| \| \| \|  \| \| --- \| \| \| --- \| --- \| \| \| \| \| \| **Table of Miss_star by size** \| \| \| \| \| \| \| --- \| --- \| --- \| --- \| --- \| --- \| \|  \| \| **size** \| \| \| **Total** \| \| **Large** \| **Mediu** \| **Small** \| \| **Miss_star** \|  \| 2025 \| 2503 \| 1504 \| 6032 \| \| **0** \| **Frequency** \| \| **Percent** \| 30.44 \| 37.62 \| 22.61 \| 90.67 \| \| **Row Pct** \| 33.57 \| 41.50 \| 24.93 \|  \| \| **Col Pct** \| 98.11 \| 94.31 \| 77.73 \|  \| \| **1** \| **Frequency** \| 39 \| 151 \| 431 \| 621 \| \| **Percent** \| 0.59 \| 2.27 \| 6.48 \| 9.33 \| \| **Row Pct** \| 6.28 \| 24.32 \| 69.40 \|  \| \| **Col Pct** \| 1.89 \| 5.69 \| 22.27 \|  \| \|  \|  \| 2064 \| 2654 \| 1935 \| 6653 \| \| **Total** \| **Frequency** \| \| **Percent** \| 31.02 \| 39.89 \| 29.08 \| 100.00 \| \| \| --- \| --- \| --- \| --- \| --- \| --- \| --- \| --- \| --- \| --- \| --- \| --- \| --- \| --- \| --- \| --- \| --- \| --- \| --- \| --- \| --- \| --- \| --- \| --- \| --- \| --- \| --- \| --- \| --- \| --- \| --- \| --- \| --- \| --- \| --- \| --- \| --- \| --- \| --- \| --- \| --- \| --- \| --- \| --- \| --- \| --- \| --- \| --- \| --- \| --- \| --- \| --- \| --- \| --- \| --- \| --- \| --- \| --- \| --- \| --- \| --- \| --- \| --- \| --- \| --- \| --- \| --- \| --- \| --- \| --- \| --- \| --- \| --- \| \| \| --- \| --- \| --- \| --- \| --- \| --- \| --- \| --- \| --- \| --- \| --- \| --- \| --- \| --- \| --- \| --- \| --- \| --- \| --- \| --- \| --- \| --- \| --- \| --- \| --- \| --- \| --- \| --- \| --- \| --- \| --- \| --- \| --- \| --- \| --- \| --- \| --- \| --- \| --- \| --- \| --- \| --- \| --- \| --- \| --- \| --- \| --- \| --- \| --- \| --- \| --- \| --- \| --- \| --- \| --- \| --- \| --- \| --- \| --- \| --- \| --- \| --- \| --- \| --- \| --- \| --- \| --- \| --- \| --- \| --- \| --- \| --- \| --- \| --- \| \| \| \| \| \| **Table of Miss_star by region** \| \| \| \| \| \| \| \| --- \| --- \| --- \| --- \| --- \| --- \| --- \| \|  \| \| **region** \| \| \| \| **Total** \| \| **Northeast** \| **South** \| **Midwest** \| **West** \| \| **Miss_star** \|  \| 893 \| 2565 \| 1493 \| 1081 \| 6032 \| \| **0** \| **Frequency** \| \| **Percent** \| 13.42 \| 38.55 \| 22.44 \| 16.25 \| 90.67 \| \| **Row Pct** \| 14.80 \| 42.52 \| 24.75 \| 17.92 \|  \| \| **Col Pct** \| 90.66 \| 90.48 \| 90.43 \| 91.46 \|  \| \| **1** \| **Frequency** \| 92 \| 270 \| 158 \| 101 \| 621 \| \| **Percent** \| 1.38 \| 4.06 \| 2.37 \| 1.52 \| 9.33 \| \| **Row Pct** \| 14.81 \| 43.48 \| 25.44 \| 16.26 \|  \| \| **Col Pct** \| 9.34 \| 9.52 \| 9.57 \| 8.54 \|  \| \|  \|  \| 985 \| 2835 \| 1651 \| 1182 \| 6653 \| \| **Total** \| **Frequency** \| \| **Percent** \| 14.81 \| 42.61 \| 24.82 \| 17.77 \| 100.00 \| \| \| --- \| --- \| --- \| --- \| --- \| --- \| --- \| --- \| --- \| --- \| --- \| --- \| --- \| --- \| --- \| --- \| --- \| --- \| --- \| --- \| --- \| --- \| --- \| --- \| --- \| --- \| --- \| --- \| --- \| --- \| --- \| --- \| --- \| --- \| --- \| --- \| --- \| --- \| --- \| --- \| --- \| --- \| --- \| --- \| --- \| --- \| --- \| --- \| --- \| --- \| --- \| --- \| --- \| --- \| --- \| --- \| --- \| --- \| --- \| --- \| --- \| --- \| --- \| --- \| --- \| --- \| --- \| --- \| --- \| --- \| --- \| --- \| --- \| --- \| --- \| --- \| --- \| --- \| --- \| --- \| --- \| --- \| --- \| --- \| --- \| --- \| \| \| --- \| --- \| --- \| --- \| --- \| --- \| --- \| --- \| --- \| --- \| --- \| --- \| --- \| --- \| --- \| --- \| --- \| --- \| --- \| --- \| --- \| --- \| --- \| --- \| --- \| --- \| --- \| --- \| --- \| --- \| --- \| --- \| --- \| --- \| --- \| --- \| --- \| --- \| --- \| --- \| --- \| --- \| --- \| --- \| --- \| --- \| --- \| --- \| --- \| --- \| --- \| --- \| --- \| --- \| --- \| --- \| --- \| --- \| --- \| --- \| --- \| --- \| --- \| --- \| --- \| --- \| --- \| --- \| --- \| --- \| --- \| --- \| --- \| --- \| --- \| --- \| --- \| --- \| --- \| --- \| --- \| --- \| --- \| --- \| --- \| --- \| --- \| \| \| \| \|  \| \| --- \| \| \| --- \| --- \| \| \| \| \| --- \| --- \| --- \| --- \| --- \| --- \| --- \| --- \| --- \| --- \| --- \| --- \| --- \| --- \| --- \| --- \| --- \| --- \| --- \| --- \| --- \| --- \| --- \| --- \| --- \| --- \| --- \| --- \| --- \| --- \| --- \| --- \| --- \| --- \| --- \| --- \| --- \| --- \| --- \| --- \| --- \| --- \| --- \| --- \| --- \| --- \| --- \| --- \| --- \| --- \| --- \| --- \| --- \| --- \| --- \| --- \| --- \| --- \| --- \| --- \| --- \| --- \| --- \| --- \| --- \| --- \| --- \| --- \| --- \| --- \| --- \| --- \| --- \| --- \| --- \| --- \| --- \| --- \| --- \| --- \| --- \| --- \| --- \| --- \| --- \| --- \| --- \| --- \| --- \| --- \| --- \| --- \| --- \| --- \| --- \| --- \| --- \| --- \| --- \| --- \| --- \| --- \| --- \| --- \| --- \| --- \| --- \| --- \| --- \| --- \| --- \| --- \| --- \| --- \| --- \| --- \| --- \| --- \| --- \| --- \| --- \| --- \| --- \| --- \| --- \| --- \| --- \| --- \| --- \| --- \| --- \| --- \| --- \| --- \| --- \| --- \| --- \| --- \| --- \| --- \| --- \| --- \| --- \| --- \| --- \| --- \| --- \| --- \| --- \| --- \| --- \| --- \| --- \| --- \| --- \| --- \| --- \| --- \| --- \| --- \| --- \| --- \| --- \| --- \| --- \| --- \| --- \| --- \| --- \| --- \| --- \| --- \| --- \| --- \| --- \| --- \| --- \| --- \| --- \| --- \| --- \| --- \| --- \| --- \| --- \| --- \| --- \| --- \| --- \| --- \| --- \| --- \| --- \| --- \| --- \| --- \| --- \| --- \| --- \| --- \| --- \| --- \| --- \| --- \| --- \| --- \| --- \| --- \| --- \| --- \| --- \| --- \| --- \| --- \| --- \| --- \| --- \| --- \| --- \| --- \| --- \| --- \| --- \| --- \| --- \| --- \| --- \| --- \| --- \| --- \| --- \| --- \| --- \| --- \| --- \| --- \| |
| --- | --- | --- | --- | --- | --- | --- | --- | --- | --- | --- | --- | --- | --- | --- | --- | --- | --- | --- | --- | --- | --- | --- | --- | --- | --- | --- | --- | --- | --- | --- | --- | --- | --- | --- | --- | --- | --- | --- | --- | --- | --- | --- | --- | --- | --- | --- | --- | --- | --- | --- | --- | --- | --- | --- | --- | --- | --- | --- | --- | --- | --- | --- | --- | --- | --- | --- | --- | --- | --- | --- | --- | --- | --- | --- | --- | --- | --- | --- | --- | --- | --- | --- | --- | --- | --- | --- | --- | --- | --- | --- | --- | --- | --- | --- | --- | --- | --- | --- | --- | --- | --- | --- | --- | --- | --- | --- | --- | --- | --- | --- | --- | --- | --- | --- | --- | --- | --- | --- | --- | --- | --- | --- | --- | --- | --- | --- | --- | --- | --- | --- | --- | --- | --- | --- | --- | --- | --- | --- | --- | --- | --- | --- | --- | --- | --- | --- | --- | --- | --- | --- | --- | --- | --- | --- | --- | --- | --- | --- | --- | --- | --- | --- | --- | --- | --- | --- | --- | --- | --- | --- | --- | --- | --- | --- | --- | --- | --- | --- | --- | --- | --- | --- | --- | --- | --- | --- | --- | --- | --- | --- | --- | --- | --- | --- | --- | --- | --- | --- | --- | --- | --- | --- | --- | --- | --- | --- | --- | --- | --- | --- | --- | --- | --- | --- | --- | --- | --- | --- | --- | --- | --- | --- | --- | --- | --- | --- | --- | --- | --- | --- | --- | --- | --- | --- | --- | --- |
| \| \| \|  \| \| --- \| \| \| --- \| --- \| \| \| \| **The MEANS Procedure** \| \| --- \| \| \| --- \| --- \| \| \| \| **Variable** \| **Label** \| **N** \| **Mean** \| **Std Dev** \| **Median** \| **Lower Quartile** \| **Upper Quartile** \| \| --- \| --- \| --- \| --- \| --- \| --- \| --- \| --- \| \| \| PercPopBelowFPL \| \| --- \| \| PercPopAA1R \| \| PercPopHisp \| \| meanMedianIncome \| \| \| % Population Below Federal Povery Level \| \| --- \| \| % Population African-American One Race \| \| % Population Hispanic Ethnicity \| \| Median Household Income $ \| \| \| 618 \| \| --- \| \| 619 \| \| 619 \| \| 618 \| \| \| 17.7626987 \| \| --- \| \| 17.3868654 \| \| 15.1621403 \| \| 53838.64 \| \| \| 10.8695191 \| \| --- \| \| 21.0525993 \| \| 19.1432162 \| \| 20989.95 \| \| \| 15.1754044 \| \| --- \| \| 8.7747090 \| \| 7.5845053 \| \| 50322.50 \| \| \| 9.4753431 \| \| --- \| \| 2.8500414 \| \| 3.2491103 \| \| 38198.00 \| \| \| 23.5203385 \| \| --- \| \| 23.3353590 \| \| 19.0922262 \| \| 65867.00 \| \| \| \| --- \| --- \| --- \| --- \| --- \| --- \| --- \| --- \| --- \| --- \| --- \| --- \| --- \| --- \| --- \| --- \| --- \| --- \| --- \| --- \| --- \| --- \| --- \| --- \| --- \| --- \| --- \| --- \| --- \| --- \| --- \| --- \| --- \| --- \| --- \| --- \| --- \| --- \| --- \| --- \| --- \| --- \| --- \| --- \| --- \| --- \| --- \| --- \| --- \| \| \| \| \| --- \| --- \| --- \| --- \| --- \| --- \| --- \| --- \| --- \| --- \| --- \| --- \| --- \| --- \| --- \| --- \| --- \| --- \| --- \| --- \| --- \| --- \| --- \| --- \| --- \| --- \| --- \| --- \| --- \| --- \| --- \| --- \| --- \| --- \| --- \| --- \| --- \| --- \| --- \| --- \| --- \| --- \| --- \| --- \| --- \| --- \| --- \| --- \| --- \| --- \| --- \| --- \| --- \| --- \| --- \| --- \| |
| \| \| \|  \| \| --- \| \| \| --- \| --- \| \| \| \|  \| \| --- \| \| \| --- \| --- \| \| \| \| **Variable** \| **Label** \| **N** \| **Mean** \| **Std Dev** \| **Median** \| **Lower Quartile** \| **Upper Quartile** \| \| --- \| --- \| --- \| --- \| --- \| --- \| --- \| --- \| \| \| PercPopBelowFPL \| \| --- \| \| PercPopAA1R \| \| PercPopHisp \| \| meanMedianIncome \| \| \| % Population Below Federal Povery Level \| \| --- \| \| % Population African-American One Race \| \| % Population Hispanic Ethnicity \| \| Median Household Income $ \| \| \| 6010 \| \| --- \| \| 6015 \| \| 6015 \| \| 6009 \| \| \| 18.2972440 \| \| --- \| \| 17.6811702 \| \| 16.7336039 \| \| 51369.18 \| \| \| 10.1767668 \| \| --- \| \| 22.1577242 \| \| 21.4920235 \| \| 20281.40 \| \| \| 16.6621419 \| \| --- \| \| 7.7372037 \| \| 7.4019726 \| \| 46772.00 \| \| \| 10.5994019 \| \| --- \| \| 2.3825587 \| \| 3.1125978 \| \| 37758.00 \| \| \| 23.8316769 \| \| --- \| \| 24.6789405 \| \| 21.0285132 \| \| 60767.00 \| \| \| \| --- \| --- \| --- \| --- \| --- \| --- \| --- \| --- \| --- \| --- \| --- \| --- \| --- \| --- \| --- \| --- \| --- \| --- \| --- \| --- \| --- \| --- \| --- \| --- \| --- \| --- \| --- \| --- \| --- \| --- \| --- \| --- \| --- \| --- \| --- \| --- \| --- \| --- \| --- \| --- \| --- \| --- \| --- \| --- \| --- \| --- \| --- \| --- \| --- \| \| \| \| \| --- \| --- \| --- \| --- \| --- \| --- \| --- \| --- \| --- \| --- \| --- \| --- \| --- \| --- \| --- \| --- \| --- \| --- \| --- \| --- \| --- \| --- \| --- \| --- \| --- \| --- \| --- \| --- \| --- \| --- \| --- \| --- \| --- \| --- \| --- \| --- \| --- \| --- \| --- \| --- \| --- \| --- \| --- \| --- \| --- \| --- \| --- \| --- \| --- \| --- \| --- \| --- \| --- \| --- \| --- \| --- \| |
