# Supplementary material for: Area-level poverty, race/ethnicity & dialysis star ratings
Source: PLoS One. 2017 Oct 17;12(10):e0186651. doi: 10.1371/journal.pone.0186651 (PMC5645143; doi:10.1371/journal.pone.0186651)
Supplement: Supplementary file 2 [file pone.0186651.s001.docx]

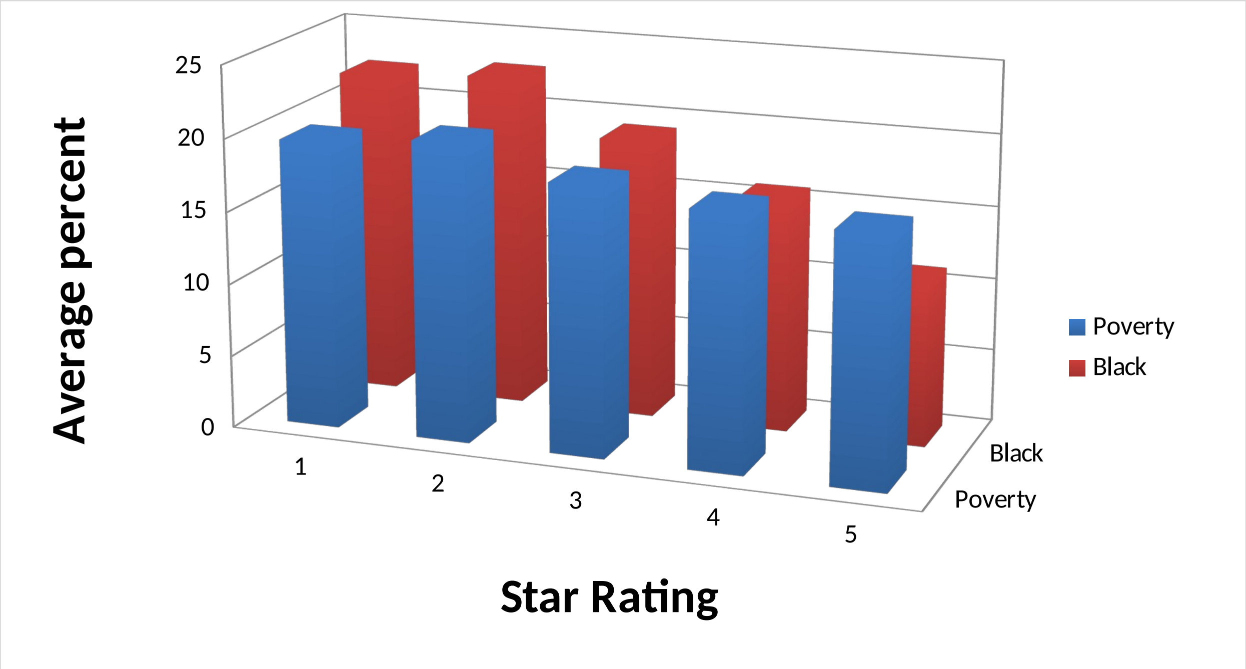
25
20
15
10
5
0
Average percent
1
2
3
4
5
Black
Poverty
Poverty
Black
Star Rating

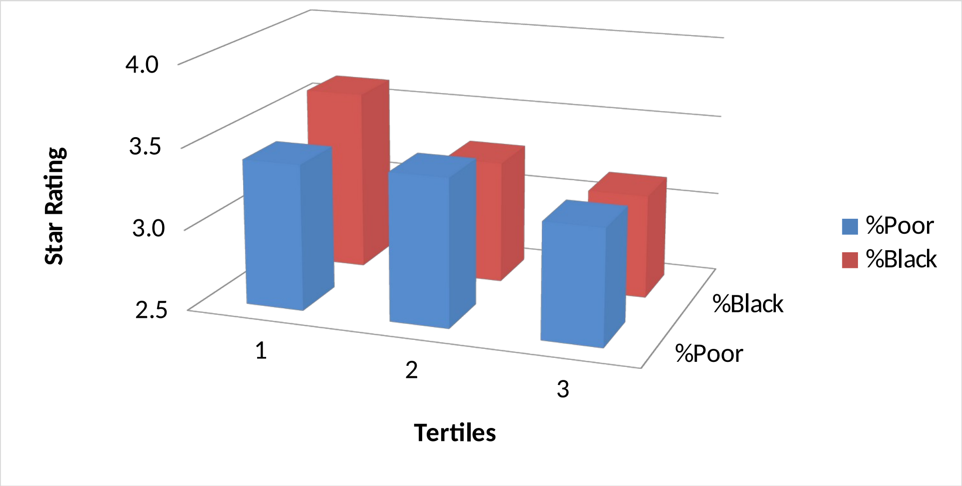
Star Rating
4.0
3.5
3.0
2.5
1
2
3
%Poor
%Black
Tertiles
%Poor
%Black

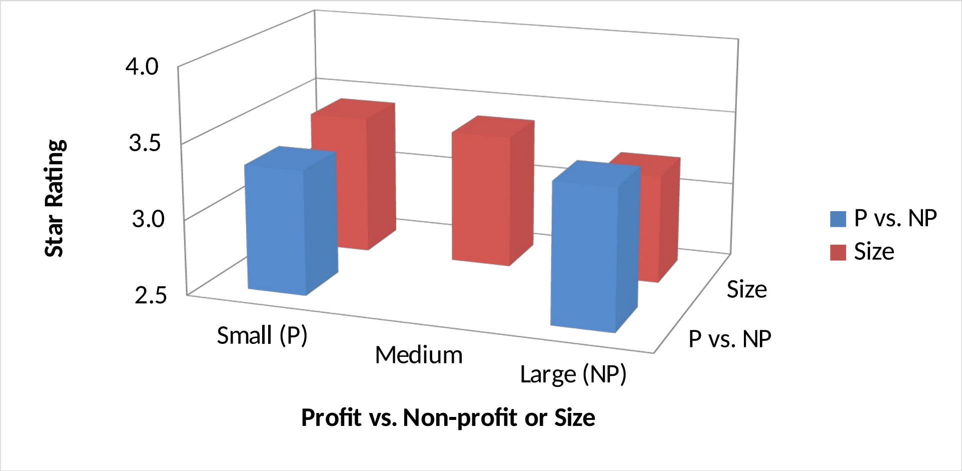
Star Rating
4.0
3.5
3.0
2.5
Small (P)
Medium
Large (NP)
P vs. NP
Size
Profit vs. Non-profit or Size
P vs. NP
Size

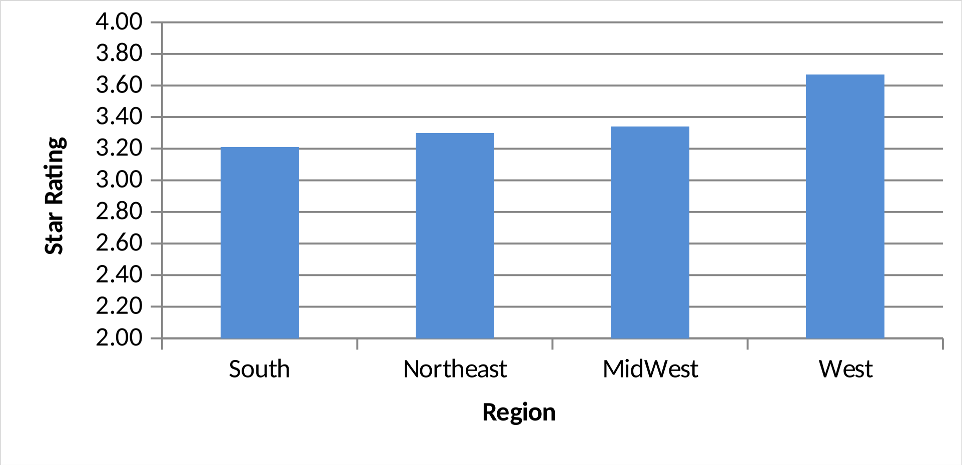
Star Rating
4.00
3.80
3.60
3.40
3.20
3.00
2.80
2.60
2.40
2.20
2.00
South
Northeast
MidWest
West
Region
